# Prognostic Value of Immunohistochemical Markers for Locally Advanced Rectal Cancer

**DOI:** 10.3390/molecules27030596

**Published:** 2022-01-18

**Authors:** Anas Taha, Stephanie Taha-Mehlitz, Stephanie Petzold, Sergey L. Achinovich, Dmitry Zinovkin, Bassey Enodien, Md Zahidul I. Pranjol, Eldar A. Nadyrov

**Affiliations:** 1 Department of Surgery, GZO Hospital, 8620 Wetzikon, Switzerland; bassey.enodien@gzo.ch; 2 Department of Biomedical Engineering, Faculty of Medicine, University of Basel, 4123 Allschwil, Switzerland; 3 Clarunis, Department of Visceral Surgery, University Centre for Gastrointestinal and Liver Diseases, St. Clara Hospital and University Hospital Basel, 4002 Basel, Switzerland; stephanie.taha@clarunis.ch; 4 Faculty of Medicine, Eberhard-Karls-University, 72074 Tübingen, Germany; stephaniepetzold@gmx.de; 5 Department of Pathology, Gomel State Medical University, 211657 Gomel, Belarus; ser.achinowitch2017@yandex.ru (S.L.A.); zinovkin2012@gmail.com (D.Z.); nadyrov2006@rambler.ru (E.A.N.); 6 School of Life Sciences, University of Sussex, Brighton BN1 9QG, UK; z.pranjol@sussex.ac.uk

**Keywords:** rectal cancer, apoptosis, proliferation, radiotherapy, Chromogranin A

## Abstract

The aim of this study is to reveal the potential roles of apoptosis markers (Bcl2 and p53), proliferation markers (Ki-67 and CyclD1), and the neuroendocrine marker Chromogranin A as markers for the radioresistance of rectal cancer. Statistically significant differences were found in the expression of p53, Ki-67, and Chromogranin A in groups of patients with and without a favorable prognosis after radiotherapy. The survival analysis revealed that the marker of neuroendocrine differentiation, Chromogranin A, also demonstrated a high prognostic significance, indicating a poor prognosis. Markers of proliferation and apoptosis had no prognostic value for patients who received preoperative radiotherapy. Higher Chromogranin A values were predictors of poor prognosis. The results obtained from studying the Chromogranin A expression suggest that the secretion of biologically active substances by neuroendocrine cells causes an increase in tumor aggressiveness.

## 1. Introduction

Over 1.8 million new colorectal cancer cases and 881,000 deaths were estimated to have occurred in 2018, accounting for about 1 in 10 cancer cases and deaths. Overall, colorectal cancer ranks third in terms of incidence but second in terms of mortality. Colorectal cancer incidence rates are about threefold higher in transitioned versus transitioning countries; however, with an average case fatality higher in lower human development index settings, there is less variation in the mortality rates. Assessing incidence and mortality trends, Arnold et al. identified three distinct global temporal patterns linked to development levels: (1) increases in both incidence and mortality rates in the most recent decade (including the Baltic countries, Russia, China, and Brazil); (2) increasing incidence but decreasing mortality (Canada, the United Kingdom, Denmark, and Singapore); and (3) both decreasing incidence and decreasing mortality (the United States, Japan, and France). The rises in incidence—particularly the generational changes detected in most age-period-cohort analyses—point to the influence of dietary patterns, obesity, and lifestyle factors, whereas the mortality declines seen in more developed countries reflect improvements in survival through the adoption of best practices in cancer treatment and management in developed countries [1,2].

Modern international standards of malignant neoplasm diagnostics provide for a comprehensive examination of tumor biopsy. The comprehensive analysis of factors involved in the maintenance of the tissue homeostasis of rectal cancer (RC) includes the evaluation of various clinical, morphological, and molecular biological parameters: longitudinal size of the tumor, age, sex, metastases, and others. Using traditional histological methods, the stage is assessed by pTNM, the degree of malignancy (G) [3].

The immunohistochemical method allows us to estimate the proliferative activity of the neoplasm by the expression of the Ki-67 proliferation marker and the factor regulating the cell cycle–CyclineD1 (CycD1), the presence of endocrine cells in the parenchymatous component of RC by the expression of Chromogranin A (ChrgA), to estimate apoptosis by the expression of the transcription factor p53 and apoptosis regulator–protein Bcl2 [4,5].

In this study, we investigate the potential roles of apoptosis markers (Bcl2 and p53), proliferation markers (Ki-67 and CyclD1), and the neuroendocrine marker ChrgA as markers for diagnosing rectal cancer. In this study, we hypothesize that apoptotic markers (Bcl2 and p53), proliferation markers (Ki-67 and CyclD1), and the neuroendocrine marker ChrgA change after radiotherapy.

Using immunohistochemistry, we examined the expression of these markers as possible determinants to evaluate the biological potential of the tumor and predict the course of RC.

## 2. Materials and Methods

### 2.1. Ethical Issues

The research protocol was registered by the Ethics Committee of Gomel State Medical University. Participants provided their informed consent, and their anonymity was maintained. An identification number was assigned to each patient throughout the observation period. After the study was completed, all patient information collected in connection with the study was stored in the archives of the Gomel Clinical Oncology Dispensary.

### 2.2. Composition and Sample Size

In this translational research, 154 rectal cancer cases were included in the study. The subjects of the study were patients who had received rectal adenocarcinomas from June 2003 to May 2017, and who were in the Abdominal Oncology Department of the Gomel Clinical Oncology Dispensary.

All patients were between 40 and 70 years of age. The median age was 63.0 (58.0–67.0) years. The ratio between men and women was 1.3/1.0. The surgical material used was 64 cases of RC I–III stage after combined treatment (preoperative radiotherapy and surgery) and 90 cases of RC I–III stage after surgical treatment (without radiotherapy). The resection edges were studied and the status of the regional lymph nodes was assessed. Preoperative radiotherapy as a component of combined treatment was performed in the preoperative period. The total dose was 25 Gy with 5 Gy dose per fraction. The operation was performed 2–3 days after the end of radiotherapy according to the approved national clinical protocols of diagnostics and treatment of malignant tumors. Clinical and morphological indices of patients with RC are presented in Table 1.

### 2.3. Data Collection Tools

For each case, relevant information on age, sex, tumor size, histological type, tumor class, stage, nodes, and metastases was collected from clinical data folders. Clinical and pathological signs were classified according to the TNM system [6].

Life expectancy of patients from the beginning of treatment was traced in all observations within the period from 1 to 168 months.

### 2.4. Histological and Immunohistochemical Studies

The materials from the tumor and the resection edges were subjected to histological and immunohistochemical examination. We studied the expression of apoptosis markers (Bcl2 and p53), proliferation markers (Ki-67 and CyclD1), and neuroendocrine marker ChrgA. The immunohistochemical reaction was made for all cases.

All pieces of tissue were fixed in 10% neutral formalin. Wax blocks were used to prepare slices 4 microns thick, followed by hematoxylin and eosin staining. The hardware-software complex Nikon was used for morphometric examination. The micropreparations were photographed with the Nikon Eclipse 50i microscope with the DS-F1 digital camera with 1689 by 1415 pixels resolution. The parameters were counted using an image analysis application package. The field of view area was 299.11 × 397.67 = 118 952.07 μm^2^ (magnification: ×400).

### 2.5. Criteria for Evaluation of Immunohistochemical Research Results

The expression of p53, CyclD1, Ki-67, BCL2, and ChrgA was determined in the tumor parenchyma. The number of positively stained cells in six non-overlapping microscope fields of view was counted (magnification: ×400).

## 3. Statistical Methods

The Mann–Whitney U test was utilized for the comparative characterization of features. The prognostic values of immunohistochemical markers (area under the ROC curve, confidence interval (CI), sensitivity, specificity, and threshold criteria) were determined using ROC analysis. The criterion of the prognostic importance of immunohistochemical markers was the presence of statistically significant differences when comparing groups using the Mann–Whitney U-criterion. In addition to analyzing the statistical significance in ROC analysis, the sensitivity index was set at > 70. The level of statistical significance was taken as *p* < 0.05. The median (Me), 25th and 75th percentiles, Me (25%, 75%), were used to represent numerical values. GraphPad Prism v.8.1 (GraphPad Software Inc., San Diego, CA, USA) and MedCalc 19.5.2 were used for analysis.

## 4. Results

The survival rate for patients with surgical treatment and combined therapy did not significantly differ (*p* = 0.743). Table 2 presents cumulative proportional survival data for all patients enrolled in the study.

The observed survival rate of patients for the three-year time interval was 71.3%, for the five-year period, 59.9%, and for the ten-year period, 52.1%, respectively. Thus, during the first three years of observation, 29.7% of patients died, five years, 41.1%, and ten years, 47.9%. Taking into consideration that the maximum growth of deceased patients occurred during the first three years of observation, this very period of observation was accepted for dividing patients into study groups.

The comparative analysis of immunohistochemical marker expression showed that in patients following preoperative radiotherapy, a poor clinical prognosis (survival rate up to three years) was associated with high expression of Ki67 proliferation marker (*p* < 0.015) and ChrgA neuroendocrine differentiation marker (*p* < 0.001) (Table 3).

The ROC analysis of Ki67 expression showed that the area under the curve was 0.681 (CI 0.552–0.792, *p* = 0.008), sensitivity was low at 52.0%, specificity and threshold criterion were 79.49% and 22.3 cells, respectively. For ChrgA, the area under the curve was 0.831 (CI 0.724–0.817, *p* < 0.001), sensitivity, specificity, and threshold criteria were 87.18%, 76.0%, and 1.9 cells, respectively (Table 3).

The expression of such markers as p53, Bcl2, and CycD1 did not differ in patients with different survival rates (*p* > 0.05). The area under the ROC curve (*p* > 0.05) for these markers was also statistically significant.

**Figure 1:**
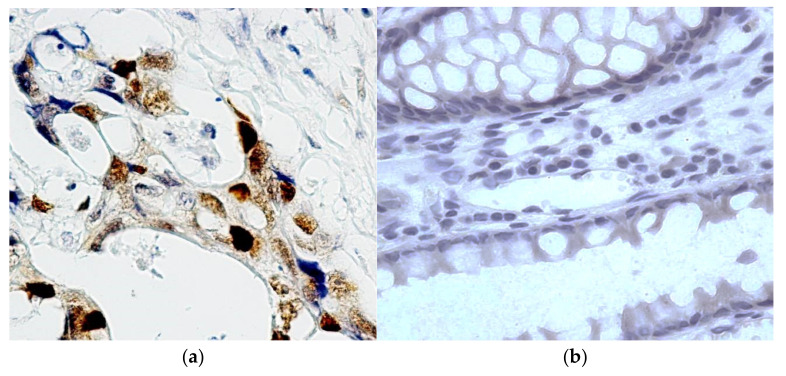
Immunohistochemical reaction with antibodies to p53 in adenocarcinoma and in the tumor resection region. (**a**): the expression of p53 is moderately expressed in RC cell nuclei, in some places sharply expressed; (**b**): the expression of p53 in the resection edge is not determined. Chromogen–diaminobenzidine. Control staining with hematoxylin staining. Magnification: ×400.

**Figure 2:**
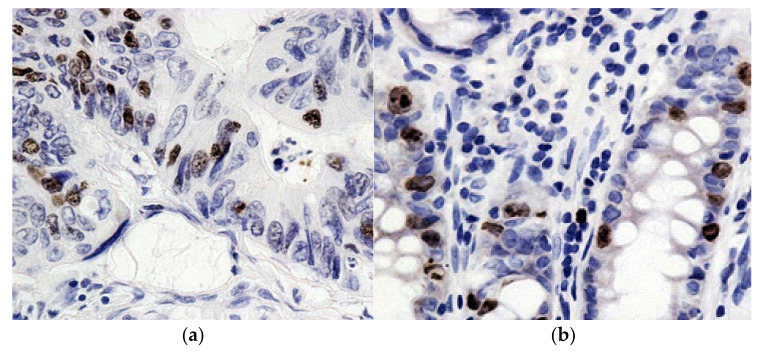
Immunohistochemical reaction with antibodies to Ki67 in adenocarcinoma and in the resection region. (**a**): moderately expressed expression of Ki67; (**b**): expression of Ki67 in the resection edge. Chromogen–diaminobenzidine. Control staining with hematoxylin staining. Magnification: ×400.

**Figure 3:**
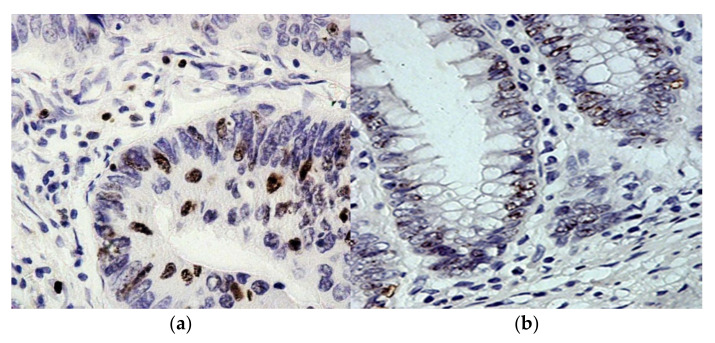
Immunohistochemical reaction with antibodies to cycline D1 in adenocarcinoma and resection region. (**a**): cycline D1 expression is determined in the nuclei of RC cells; (**b**): cycline D1 expression in colonocytes in crypts at the resection edge. Chromogen–diaminobenzidine. Control of hematoxylin staining. Magnification: ×400.

**Figure 4:**
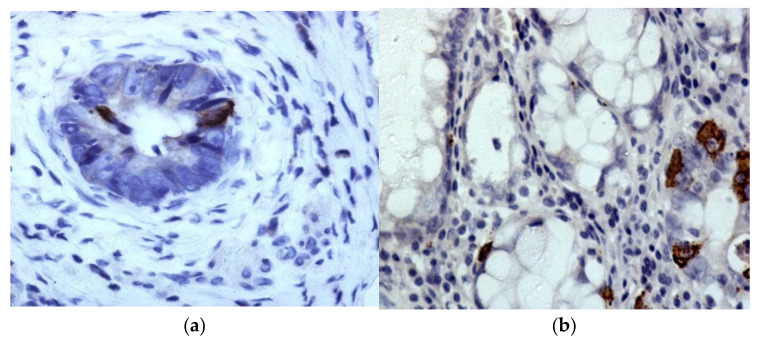
Immunohistochemical reaction with ChrgA antibodies in adenocarcinoma cells and in the resection region. (**a**): ChrgA expression in RC; (**b**): moderate and weakly expressed specific coloring is determined in the resection edge. Chromogen–diaminobenzidine. Control staining with hematoxylin staining. Magnification: ×400.

Examples of the expression of immunohistochemical markers in RC tissue are presented in Figure 1, Figure 2, Figure 3 and Figure 4.

In patients following surgical treatment (without radiotherapy), a poor clinical prognosis was associated with high expression of proliferation markers, apoptosis, and the neuroendocrine differentiation of tumor cells. For instance, the statistical significance was *p* < 0.001 for Ki67, p53, and ChrgA, and *p* = 0.018 for Bcl2. No statistically significant differences (*p* > 0.05) were found for CycD1 proliferation marker (Table 3).

The ROC analysis of Ki67 expression showed that the area under the curve was 0.867 (CI 0.775–0.941.0, *p* < 0.001), sensitivity, specificity, and threshold criteria were 84.5%, 64.52%, and 39.8 cells, respectively. For p53, the area under the curve was 0.815 (CI 0.719–0.889, *p* < 0.001), sensitivity, specificity, and threshold criteria were 89.80%, 74.19%, and 31.6 cells, respectively. For ChrgA, the area under the curve was 0.787 (CI 0.540–0.889, *p* < 0.001), sensitivity, specificity, and threshold criteria were 100%, 50.4%, and 7.4 cells, respectively. No statistically significant differences were found for CycD1 proliferation marker (*p* > 0.05).

## 5. Discussion and Conclusions

The prognostic value of markers of proliferation and apoptosis for RC has already been reported. A number of observations have shown an association between positive p53 and the early development of relapses and distant metastases in patients with RC [7,8]. No association has been observed between p53 expression and clinical and morphological parameters such as age, gender, tumor size, and disease stage [9,10]. For example, in patients following preoperative radiotherapy, this marker used as a predictor of poor prognosis did not differ when compared between groups with different survival rates (*p* = 0.110). However, patients with surgical treatment demonstrated a low survival rate and were associated with high levels of p53 expression (*p* < 0.001). In addition, the ROC analysis showed a low sensitivity of this marker (49.50%) in patients who received preoperative radiotherapy, whereas for patients who received surgery, this showed a high sensitivity (84.75%).

Bcl2 protein, a marker for apoptosis, is involved in the regulation of apoptosis. There are reports that the overexpression of Bcl2 in the parenchymal structures of colorectal cancer is associated with a more favorable prognosis [11,12]. Other authors have shown that Bcl2 expression in colorectal cancer tissue is not associated with survival [13].

According to multivariate analysis, the tumor stage, male gender of patients, and p53 and Bcl2 expression were independent predictors of RC relapse [14]. Other studies have shown a direct relationship between Bcl2 expression and neuroendocrine tumor differentiation. This indicates that additional research is needed to fully establish the role of Bcl2 as an independent prognostic factor and in combination with other markers [15]. Our research has shown that the expression of this marker is not associated with survival. In the group of patients with radiotherapy, there were no differences in Bcl2 expression in patients with survival rate of more or less than three years (*p* = 0.335). The ROC analysis also showed no prognostic significance (*p* = 0.358) for patients after preoperative radiotherapy. For patients who only had surgical treatment, the expression of this marker was lower, with a survival rate of less than three years (*p* = 0.018). The area under the ROC curve was 0.753 (0.651–0.838), statistically significant (*p* < 0.001), but the sensitivity index was very low, at 52.54%. Thus, we suggest that Bcl2 may not be a predictor of poor prognosis for RC.

To assess proliferative activity, the molecular markers Ki-67 and CycD1 have been utilized. Ki-67 is expressed in all phases of the cell cycle with the exception of G0. The results of studies on the effect of Ki-67 levels in colorectal cancer structures on the outcome of this disease are ambiguous. According to some authors, there was no correlation between Ki-67 expression and disease prognosis [16,17]. Other researchers note a link between a high proliferation index in colorectal cancer structures, determined by Ki-67 expression, and good patient survival rates [18]. The study of CycD1 expression is also used for RC prediction. This marker controls the entry of cells into the synthetic phase of the cell cycle. The effect of CycD1 expression in the prediction of RC remains ambiguous. According to some authors, patients with colorectal cancer demonstrate an increase in the overall survival rate in the presence of the overexpression of CycD1 compared to the absence of this marker in primary tumor cells [19]. Another report shows that CycD1 is not an independent prognostic sign after radical surgical treatment of colorectal cancer [20]. The results of the multivariate analysis showed that CycD1 is an independent indicator of poor prognosis in colorectal cancer [21,22]. In our study, the expression of the Ki67 proliferation marker was significantly higher with a survival rate of less than three years in both patients after radiotherapy (*p* = 0.015) and surgical treatment (*p* < 0.001). The ROC analysis showed a high specificity (84.75%) only for patients following surgical treatment. However, for patients following radiotherapy, the sensitivity was very low (52.00%), which suggest that Ki67 expression may not be a predictor of low survival rate in patients after preoperative radiotherapy.

Many studies have shown the prognostic value of markers of proliferation and apoptosis for RC. A number of observations have shown an association between positive p53 and the early development of relapses and distant metastases in patients with RC [19,20]. There is evidence that there was no association between p53 expression and such clinical and morphological parameters as age, gender, tumor size, and disease stage [10,11]. In addition, the ROC analysis showed a low sensitivity of this marker (49.50%). For patients with surgical treatment, a low survival rate was associated with high levels of p53 expression (*p* < 0.001), while the ROC analysis showed a high sensitivity (84.75%) of this marker.

To assess the prognosis of colorectal cancer, the neuroendocrine differentiation marker ChrgA has been studied. Reports have shown that the expression of ChrgA in colorectal cancer (CRC) was an indicator of poor prognosis. There are reports that ChrgA expression in CRC cells correlates with the degree of tumor differentiation, disease stage, and low survival rates. There are also reports that there was no correlation between the expression of ChrgA in CRC and the degree of differentiation of the tumor, the stage of the disease, the depth of invasion, and the localization of the neoplasm, but it was found that neuroendocrine differentiation is an independent prognostic factor that worsens survival in stage III–IV RC adenocarcinoma [5,23]. Our study confirmed reports of a high prognostic value of this marker not only in surgical treatment, but also in patients after radiotherapy. The area of the ROC curve for ChrgA in patients following radiotherapy of lymphocytes was high 0.831 (0.724–0.817, *p* < 0.001), while the sensitivity also showed high values (87.18%). Similar results were observed in patients following radiotherapy. The area of the ROC curve was 0.787 (0.540–0.889, *p* < 0.001), and the sensitivity was the highest among other indicators (100%).

Thus, our study showed that markers of proliferation and apoptosis in rectal adenocarcinoma tissue may have a prognostic value. However, this type of treatment has an impact on the prognostic significance of markers of disease prognoses. For patients who received surgical treatment only (without radiotherapy), the prognostic markers of poor prognosis were the high expression of the Ki-67 proliferation marker and P53 apoptosis. The marker of neuroendocrine differentiation, ChrgA, also demonstrated a high prognostic significance, indicating a poor prognosis. The markers of proliferation and apoptosis had no prognostic value for patients who received preoperative radiotherapy. Higher ChrgA values were predictors of poor prognosis. The results obtained from studying the ChrgA expression suggest that the secretion of biologically active substances by neuroendocrine cells causes an increase in tumor aggressiveness. It is highly likely that the inhibition of ChrgA expression after radiotherapy may be an independent criterion for the effectiveness of this type of treatment. However, this needs to be investigated further in larger patient cohorts, and the underlying molecular mechanisms ought to be assessed in order to develop novel immunotherapeutic strategies.

## Figures and Tables

**Table 1:** Characteristics of patients with RC.

Parameters	Absolute Values	RC after Combined Treatment	RC after Surgical Treatment
Number of patients	154	64	90
Age (years)	61.58 (41.15–69.25)	59.87 (42.18–68.45)	62.87 (41.63–69.89)
Survival rate less than 1 year till the age of 3 till the age of 5 over 5 years	8 30 42 74	5 14 15 30	3 16 27 44
Grade G1 G2 G3	6 134 14	3 54 7	3 80 7
Stage I TNM T1,T2 N0M0	10	3	7
Stage IIA TNM T3 N0M0	63	24	39
Stage IIB TNM T4a,b N0M0	33	11	22
Stage IIIA TNM T1,2 N1a,bM0	4	2	2
Stage IIIB TNM T3,4a N1a,bM0	39	22	17
Stage IIIC TNM T1,2,3,4a N2aM0	5	2	3

Note: all patients with stage II–III who did not have radiotherapy had contraindications.

**Table 2:** Cumulative proportional survival of patients.

Period Surveillances	Cumulative Survival Rate (%)	Standard Error	Confidence Interval
3 years	71.3	3.71	63.96–78.64
5 years	59.9	4.02	52.1–67.7
10 years	52.1	4.2	43.9–60.3

**Table 3:** Indicators of immunohistochemical markers in patients with and without preoperative radiotherapy (survival rate).

Marker	Radiotherapy		P_1_	Without Radiotherapy		P_2_
	Less than 3 Years	More than 3 Years		Less than 3 Years	More than 3 Years	
p53	51.40 [28.00; 61.50]	28.70 [8.80; 54.60]	0.110	40.80 [27.60; 51.60]	11.50 [5.20; 22.40]	<0.001
Bcl2	3.00 [1.00; 5.50]	3.40 [1.70; 6.80]	0.335	2.80 [2.30; 4.50]	6.40 [5.45; 10.65]	0.018
Ki-67	22.80 [8.20; 39.30]	8.80 [2.00; 21.50]	0.015	36.50 [15.40; 48.20]	15.70 [8.60; 29.90]	<0.001
CyclD1	6.40 [5.45; 10.65]	9.35 [5.20; 15.20]	0.956	7.80 [3.30; 28.30]	6.08 [3.80; 15.70]	0.905
ChrgA	3.00 [2.00; 6,00]	1.40 [1.10; 1.70]	<0.001	8.50 [2.00; 10.50]	1.80 [1.30; 3.50]	<0.001

Note: P_1_: statistical significance of differences in the group of patients with preoperative radiotherapy; P_2_: statistical significance of differences in the group of patients with surgical treatment (without radiotherapy).

## Data Availability

Not applicable.

## Sample Availability

Samples of the compounds are available from the authors for reasonable request.
